# Psychiatric Characteristics, Symptoms of Insomnia and Depression, Emotion Regulation, and Social Activity among Swiss Medical Students

**DOI:** 10.3390/jcm13154372

**Published:** 2024-07-26

**Authors:** Jonas Regli, Dena Sadeghi-Bahmani, Viola Rigotti, Zeno Stanga, Ismail I. Ülgür, Christian Fichter, Undine E. Lang, Annette B. Brühl, Serge Brand

**Affiliations:** 1 Faculty of Medicine, University of Basel, 4001 Basel, Switzerland; 2 Department of Psychology, Stanford University, Stanford, CA 94305, USA; bahmanid@stanford.edu; 3 Department of Epidemiology & Population Health, Stanford University, Stanford, CA 94305, USA; 4 Outpatient Medical Clinic, University Hospital of Basel, 4031 Basel, Switzerland; viola.rigotti@usb.ch; 5 Centre of Competence for Military and Disaster Medicine, Swiss Armed Forces, 3008 Bern, Switzerland; zeno-giovanni.stanga@vtg.admin.ch (Z.S.); ismail.uelguer@vtg.admin.ch (I.I.Ü.); 6 Division of Diabetes, Endocrinology, Nutritional Medicine and Metabolism, University Hospital, University of Berne, 3012 Berne, Switzerland; 7 Department of Psychology, Kalaidos University of Applied Sciences, 8050 Zurich, Switzerland; christian.fichter@kalaidos-fh.ch; 8 Adult Psychiatric Hospital of the University of Basel, 4002 Basel, Switzerland; undine.lang@upk.ch; 9 Center for Affective, Stress and Sleep Disturbances, Psychiatric Clinics of the University of Basel, 4002 Basel, Switzerland; annette.bruehl@upk.ch; 10 Division of Sport Science and Psychosocial Health, Department of Sport, Exercise and Health, Faculty of Medicine, University of Basel, 4052 Basel, Switzerland; 11 Sleep Disorders Research Center, Kermanshah University of Medical Sciences, Kermanshah 6719851115, Iran; 12 Substance Abuse Prevention Research Center, Health Institute, Kermanshah University of Medical Sciences, Kermanshah 6719851115, Iran; 13 School of Medicine, Tehran University of Medical Sciences, Tehran 1417653761, Iran; 14 Center for Disaster Psychiatry and Disaster Psychology, Psychiatric Clinics of the University of Basel, 4002 Basel, Switzerland

**Keywords:** medical students, depression, insomnia, emotion regulation, social activity

## Abstract

**Background**: Almost by default, young adult students are at increased risk of suffering from mental health issues, and this holds particularly true for medical students. Indeed, compared to the general population and non-medical students, medical students report higher scores for symptoms of depression. For Swiss medical students, research on the associations between psychiatric characteristics and symptoms of depression and insomnia, including cognitive–emotional processes and social activity, has been lacking so far. Given this, the aims of the present study were to relate self-declared psychiatric characteristics to symptoms of depression, insomnia, emotion regulation, and social activity. **Methods**: A total of 575 medical students (mean age: 22.4 years; 68.9% females) completed an online survey covering sociodemographic information (age and gender), study context (year of study), self-declared psychiatric characteristics and symptoms of depression, insomnia, emotion regulation (cognitive reappraisal vs. emotion suppression), and social activity. Data on insomnia sum scores and categories of historical samples (862 non-medical students and 533 police and emergency response service officers) were used for comparison. **Results**: Of the 575 participants, 190 participants (33%) self-declared psychiatric issues, such as major depressive disorder; anxiety disorders, including PTSD and adjustment disorders; eating disorders; ADHD; or a combination of such psychiatric issues. Self-reporting a psychiatric issue was related to higher symptoms of depression and insomnia and lower symptoms of social activity and cognitive reappraisal (always with significant *p*-values and medium effect sizes). Compared to historical data for non-medical students and police and emergency response service officers, medical students reported higher insomnia scores. In a regression model, current self-declared psychiatric issues, female gender, higher scores for insomnia, and lower scores for social activity were associated with higher scores for depression. **Conclusions**: Among a sample of Swiss medical students, the occurrence of self-declared psychiatric issues was associated with higher scores for depression and insomnia and lower cognitive reappraisal and social activity. Further, insomnia scores and insomnia categories were higher when compared to non-medical students and to police and emergency response service officers. The data suggest that medical schools might introduce specifically tailored intervention and support programs to mitigate medical students’ mental health issues. This holds particularly true for insomnia, as standardized and online-delivered treatment programs for insomnia (eCBTi) are available.

## 1. Introduction

Compared to adolescence and middle and late adulthood, early adulthood is a particularly demanding developmental stage of life, as individuals in this age range have to cope with challenging vocational and psychosocial developmental tasks [1,2]. Thus, for this age range, the pressure to be successful is particularly high, and, unsurprisingly, suicide rates are highest among 18-to-25-year-old individuals in general and among males in particular [3]. 

### 1.1. Mental Health Issues in the General Population

For the global general adult population, the WHO Mental Health Report [4] indicated that by 2019, that is, before the COVID-19 pandemic and its psychosocial restrictions, about 13% had mental health issues, and about 14.6% of mental health issues were observed among young adults aged 20 to 24 years. Most critically, before the COVID-19 pandemic, increasing incidence rates of 28% for symptoms of anxiety and 26% for symptoms of depression were observed. 

For Switzerland, the recent report of the OBSAN (Observatoire Suisse de Santé, 2023; [5]) reported data gathered in 2022. Briefly, self-declared symptoms of depression were reported as follows: 35.9% for the whole population, 65.1% among females aged between 15 and 24 years (with 30.4% reporting moderate to severe symptoms of depression), 50% among females aged 25 to 35 years (with 18.6% reporting moderate to severe symptoms of depression), and 41.3% among males aged between 15 and 24 years (with 15% reporting moderate to severe symptoms of depression). Further, 16.5% reported suffering from symptoms of anxiety within the last 12 months, with prevalence rates of self-declared symptoms of generalized anxiety disorders of 32.1% and 48% among 15–24-year-old persons and 41.3% among 25-to-34-year-old persons. Prevalence rates of suicidal ideation were 8.3% within the last two weeks and 5% within the last 12 months. 

Overall, self-declared symptoms of psychiatric issues appeared to be particularly high among the Swiss general population, and peaks were observed for individuals in late adolescence and early adulthood and among females. Please note that the definition of emerging or young adulthood between 18 and 25 years may vary between disciplines of medicine or psychology and authors [6].

### 1.2. Mental Health Issues among Students and Medical Students

A further subgroup of the general population reporting higher mental health issues is young adult students in general [7,8,9,10,11,12,13,14,15,16,17,18,19,20,21,22,23,24,25,26,27,28] and medical students in particular [13,14,17,18,25,26,29].

To illustrate, two comprehensive meta-analyses reported that 27.2% [30] to 28% [31] of medical students suffered from symptoms of depression, with a range from 9 to 55% [30]. Along with symptoms of depression, symptoms of burnout, suicidal thoughts, and anxiety disorders were also observed [27,28,32,33]. To explain such high prevalence rates of symptoms of mental health issues, stress related to the study of medicine appeared to be the main driver [13,34,35]. Next, differences in study designs, including assessment measures, study curricula [36], and countries [37] appeared to further explain the broad variety of prevalence rates for symptoms of depression, while within-country variations were also observed. To make the case in point, for symptoms of depression, medical students of the University of Munich (Germany) reported prevalence rates of 28%, while medical students of the University of Witten (Germany) reported a prevalence rate of 15% [36], showcasing intra-country variability (for Germany: [18,25,36,38]; for Portugal: [35]; for Sweden: [34]; and for Norway [39]).

If we consider symptoms of social anxiety as the opposite pole of social activity, for medical students, the following observations were reported: Hazmi, et al. [40] reported a prevalence rate of 13.5% among Saudi medical students, while Alkhalifah, et al. [41] observed prevalence rates of up to 61.7% among female medical students and about 38.3% among male medical students. While such prevalence rates were reported from a specific cultural area (Saudi Arabia), one might assume that similar prevalence rates might also be observed in other regions among medical students. While this was not the focus of the present study, Quek, et al. [28] showed in their meta-analysis covering 40,398 medical students included in 69 studies a prevalence rate of 33.8% for anxiety disorders. Given these findings, and again considering anxiety and social anxiety as the opposite pole of social activity, it appeared plausible to observe issues regarding social anxiety among the present sample.

For Switzerland, Ernst, et al. [14] reported a prevalence of clinically relevant depressive symptoms of 27.2% among medical students, though more reliable data appear to be needed, as the study was conducted during the COVID-19 pandemic and its social restrictions and it included only about 9% of eligible medical students. 

Overall, young female adults in general and students and medical students in particular are at increased risk of reporting higher rates of mental health issues. Given this, the first aim of the present study was to assess the prevalence rates of self-declared psychiatric issues among Swiss medical students and to investigate whether the self-declared occurrence of psychiatric issues was related to higher scores for psychological ill-being. 

### 1.3. Symptoms of Depression and Insomnia 

Almost by nature, individuals reporting symptoms of depression also report poor sleep, and there is now sufficient and robust evidence from cross-sectional studies and meta-analytical reviews that poor sleep and symptoms of depression are highly associated [42,43,44,45,46,47,48,49,50,51,52,53,54,55]. Further, and most importantly, meta-analyses and systematic reviews summarizing results from longitudinal data evidenced that poor sleep was the causal factor for the onset of symptoms of depression [48,49,56] and that cognitive–behavioral therapy (CBT)-based interventions to treat insomnia also favorably impacted on symptoms of depression [45]. Given this, the next aim of the present study was to investigate the degree of association between symptoms of depression and insomnia.

There is sufficient evidence that compared to the general population and to non-medical students, medical students are at increased risk of reporting higher scores for insomnia [57,58,59], though it appeared that no such data were available for medical students in Switzerland. To counter this, a further aim of the present study was to assess symptoms of insomnia to compare the intensity and insomnia categories with those of two historical samples (non-medical students and police and emergency response service officers) and to relate medical students’ insomnia scores to emotion regulation and social activity.

### 1.4. Emotion Regulation, Symptoms of Depression, and Insomnia

Intuitively and following evidence-based research, poor sleep and symptoms of depression are associated with dysfunctional cognitive and emotional processes [60]. Regarding poor sleep, among other factors, dysfunctional pre-sleep cognitions [61,62,63,64,65,66,67,68,69] and stress [70,71,72,73] were identified as possible causes, including cognitive and mental hyperarousal [74,75]. Further, poor sleep patterns predicted internalizing problems in later life [76,77,78], while poor sleep was also associated with higher scores for emotional reactivity [79] and impulsivity [80]. Similarly, higher scores for symptoms of depression were associated with stress [13,34,81,82,83,84,85,86,87,88,89,90,91]. Classically, the perception of stress and coping with stress are highly cognitive–emotional processes [92], and the logic is that coping is associated with emotion regulation, that is, with cognitive reappraisal and emotion suppression [93,94]. More specifically, previous studies showed that cognitive reappraisal was associated with lower scores for insomnia and that emotion suppression was associated with higher scores for insomnia [95,96,97,98]. A plausible mechanism to explain this phenomenon is that a person’s tendency to internalize problems may lead to higher levels of emotional arousal and thus to more impaired sleep [44,75]. Thus, given that cognitive–emotional processes are highly involved in the emergence and maintenance of poor sleep, in the present study, we paid particular attention to two cognitive processes (cognitive reappraisal and emotion suppression) in relation to insomnia alongside symptoms of depression and social activity. Given this, the next aim of the present study was to investigate whether and to what extent the dimensions of cognitive reappraisal and emotion suppression were associated with symptoms of depression, insomnia, and social activity, and whether participants with and without psychiatric issues differed in emotion regulation. 

### 1.5. Perceived Social Support, Social Activity, and Symptoms of Depression

Human beings are social beings, and, from an evolutionary point of view, neocortical increase in brain volume and humans’ relatively larger neocortical size compared to that of our evolutionarily closest kin, chimpanzees, gorillas, bonobos, and orangutans, are understood to indicate that the human brain is adapted to cope with the social environment, and particularly with the social group size [99,100,101,102]. Given this, intuitively, the quality and quantity of social interactions should be crucial for a person’s mental health. Indeed, and more specifically for the general population, individuals suffering from major depressive disorder are at dramatically increased risk of reporting fewer and qualitatively more modest social interactions. To make the case in point, Ding, et al. [103] observed among individuals with chronic major depressive disorder that perceived chronic social adversities appeared to trigger and maintain symptoms of stress along with depression and anxiety. In the same vein, individuals with major depressive disorders had more difficulties differentiating between faces displaying positive emotions and those showing no emotional content [104]. Visted, et al. [105] summarized in their systematic review and meta-analysis that people with depressive episodes in their biographies (current or remitted states) also indicated having more difficulties with emotion regulation than people without such periods of life. More dramatically, these difficulties in emotion regulation appeared to persist even when symptoms of depression decreased. Accordingly, Visted, et al. [105] speculated that poor emotion regulation and more limited social competencies might be latent risk factors for relapses of depressive episodes. Further, symptoms of depression were related to feelings of loneliness as a proxy of social detachment and alienation, and scores for loneliness were higher among young adults compared to other age groups [106]. By contrast, there is sufficient evidence that perceived [107,108] or even subliminally presented social support [109] was associated with more attenuated cardiovascular reactivity scores during an acute stress paradigm. Further, at least among adolescents, social support—understood as the opposite of loneliness—was associated with higher scores for mental health [110]. In line with this, the quality of social relationships appeared to be an active ingredient in reducing symptoms of depression and anxiety among young people aged 14 to 24 years [111]. Given this, the next aim of the present study was to explore whether and to what extent an individual’s social activity was related to psychiatric issues and to symptoms of depression, insomnia, and emotion regulation.

### 1.6. The Present Study

The background to this study was as follows: Compared to the general population and non-medical students, medical students reported more mental health issues [13,14,17,18,25,26,29]. Such mental health issues are related to symptoms of depression [30,31] and insomnia [57,58,59,112]. Next, emotion regulation is a cognitive–emotional process for coping with emotions and with stress more broadly [93,94,113]. Lastly, social support and social activity appeared to be protective factors against symptoms of depression and loneliness [106,110,111], at least among individuals with major depressive disorder and among adolescents. 

However, it appeared that the above-mentioned dimensions had not been thoroughly and systematically assessed among Swiss medical students, despite the fact that medical students are at increased risk of suffering from psychiatric issues [30,31]. To counter this, we approached a larger sample of Swiss medical students of the University of Basel (Basel, Switzerland) and asked them to complete an online questionnaire on psychiatric health issues, symptoms of depression, insomnia, emotion regulation, and social activity.

The following five hypotheses and one research question were formulated.

First, we hypothesized that (a) prevalence rates of self-reported psychiatric issues would be descriptively comparable to prevalence rates reported elsewhere [30,31] and that (b) participants who self-reported psychiatric issues would also report higher scores for symptoms of depression and insomnia, higher scores for emotion suppression and lower scores for cognitive reappraisal as proxies of emotion regulation, and lower scores for social activity. Second, following others [43,44,45,46,48,49,114], we assumed that symptoms of insomnia and depression would be associated. Third, following others [57,58,59], we assumed that medical students would report higher insomnia scores (total sum and categories) compared to non-medical students and adult professionals exposed to a higher degree of danger (i.e., police officers). Fourth, using the concept of emotion regulation [94,115,116,117], we hypothesized that (a) a higher degree of cognitive reappraisal would be associated with lower scores for insomnia and depression and with higher scores for social activity, while (b) higher scores for emotion suppression would be associated with higher scores for insomnia and depression and lower scores for social activity. With the fifth hypothesis, we assumed that, based on previous results [106,110,111], higher scores for social activity would be associated with lower scores for insomnia and depression.

The research question was the following: Which dimensions (insomnia, cognitive reappraisal, emotion suppression, social activity, and occurrence of psychiatric issues) were more strongly associated with symptoms of depression in a regression model?

## 2. Methods and Measures

### 2.1. Procedure

All medical students of the University of Basel (Switzerland) were invited to take part in the present cross-sectional and anonymous online study, which was run with Tivian^®^/Questback^®^. On the first page of the online study, participants were fully informed about the aims of the study, the anonymous data gathering, and the anonymous data elaboration. Participants were also informed that participation or non-participation had no advantages or disadvantages for the continuation of their medical studies and that they could stop or interrupt their participation at any time. The first page of the online study further indicated a series of phone numbers, email addresses, and precise locations of local mental healthcare centers in the event that a person needed or wanted further help for their possible mental health issues. Next, ticking the box “I have read and understood the study conditions, and I agree to participate at the study” equaled signing written informed consent. Afterwards, participants completed a series of self-rating questionnaires covering sociodemographic information, self-declared psychiatric health issues, symptoms of depression, insomnia, emotion regulation, and social activity (see details below). On average, participants needed about 20 min to complete the online questionnaires. The study lasted from 24 October 2022 to 31 December 2022. The ethical committee (EKNZ; Ethikkommission Nordwest und Zentralschweiz; Basel, Switzerland; ethical code: AO_2022_00055; date of approvement: 13 October 2022) approved the study, which was performed in accordance with the seventh and current revision [118] of the Declaration of Helsinki. 

### 2.2. Participants

The inclusion criteria were as follows: (1) aged 18 years or older; (2) registered as a medical student at the University of Basel (Basel, Switzerland) or registered as a student of dental medicine within the first two years; (3) compliance with the study’s requirements; (4) “signed” written informed consent. The exclusion criterion was the following: (5) “click-throughs” who needed less than five minutes to complete the questionnaires.

The link to the online survey was accessed 1027 times. A total of 654 people gave their informed consent. Of those, 575 (87.9%) completed the survey.

Historical data on the Insomnia Severity Indices (ISIs) of 862 non-medical students (mean age: 24.3 years; 25.9% males) and 533 police officers (mean age: 41.2 years; 77.1% males) [119] were used to compare Insomnia Severity Index (ISI) scores between medical students and healthy adult populations. The historical samples consisted of adults aged 18 years and older with no self-declared signs of somatic or psychiatric symptoms. 

### 2.3. Measures

#### 2.3.1. Sociodemographic and Study-Related Information

Participants reported on their age (years), gender (male, female, or diverse), study year (bachelor or master level), and study subject (medicine or dentistry).

#### 2.3.2. Health-Related Information

Participants were asked to indicate whether they were currently suffering from a self-rated psychiatric issue (yes or no). If the answer was yes, participants were asked to specify their assumed mental health issue: depression; bipolar disorder; adjustment disorder; anxiety disorder, including panic attacks, social anxiety, generalized anxiety disorder, and PTSD; ADHD; autism spectrum disorder; substance use disorder; or eating disorder, including the co-occurrence of two or three psychiatric health issues. 

Note that, after this paragraph, participants were provided once again with a series of phone numbers, email addresses, and precise locations of local mental healthcare centers in the city of Basel (Basel, Switzerland) in the event that a participant needed or wanted further help for their possible mental health issues.

#### 2.3.3. Symptoms of Depression

To assess symptoms of depression, participants completed the German version [120] of the depression module of the Patient Health Questionnaire (PHQ), a validated tool widely used to screen for depression [121,122,123]. They were asked how often in the past two weeks they had been bothered by various problems like “Feeling down, depressed, or hopeless”, “Feeling bad about yourself—or that you are a failure or have let yourself or your family down” or “Thoughts that you would be better off dead or of hurting yourself in some way”. Answers were given on four-point scales from 0 (=not at all) to 3 (=nearly every day). A higher sum score reflected a higher intensity of symptoms of depression (Cronbach’s alpha: 0.87). 

#### 2.3.4. Insomnia

To assess insomnia, participants completed the German version [119] of the Insomnia Severity Index (ISI) [124]. It includes seven questions about sleep quality and insomnia, and the participants answered how often certain conditions concerning sleep quality had occurred during the last month on scales ranging from 0 (=never/not at all) to 4 (=always). The total score ranges from 0 to 28, with a higher sum score reflecting a higher severity of insomnia (Cronbach’s alpha: 0.90). Categories are as follows: 0–7 = no clinically significant insomnia; 8–14 = subthreshold insomnia; 15–21 = clinical insomnia (moderate severity); 22–28 = clinical insomnia (severe).

#### 2.3.5. Emotion Regulation

To asses emotion regulation, participants completed the German version [125] of the emotion regulation questionnaire (ERQ) [126]. More specifically, two strategies of emotion regulation were assessed: cognitive reappraisal and emotion suppression. For cognitive reappraisal, typical items included “When I want to feel more *positive* emotion (such as joy or amusement), I *change what I’m thinking about*” and “When I’m faced with a stressful situation, I make myself *think about* it in a way that helps me stay calm”. For emotion suppression, typical items included “I keep my emotions to myself” and “I control my emotions by *not expressing them*”. Answers were given on seven-point Likert scales ranging from 1 (=strongly disagree) to 7 (=strongly agree), with higher sum scores for cognitive reappraisal reflecting a higher tendency to cognitively reappraise both negative and positive emotions; higher scores for emotion suppression reflected a higher tendency to suppress both negative and positive emotions (Cronbach’s alphas: cognitive reappraisal: 0.91; emotion suppression: 0.89). 

#### 2.3.6. Social Activity

To assess social activity, participants completed the Social Adaption Self-Evaluation Scale (SASS) [127]. It consists of 21 items. Typical items include “How often do you see your relatives (e.g., partner; parents; siblings)?”—never (0), seldom (1), often (2), very often (3); “Besides the family members, you are in touch with…”—nobody (0), a very few people (1), some people (2), a lot of people (3)”; and “The quality of relationship with others is...?”—very unsatisfying (0), unsatisfying (1), somewhat satisfying (2), very satisfying (3)”. As shown, answers are given on 4-point Likert scales, ranging from 0 to 3, with higher sum scores reflecting higher and more satisfying social activity. The SASS has been shown to be useful and reliable in evaluating the social functioning of patients with depression [128].

### 2.4. Statistical Analysis

To answer the first hypothesis (a), prevalence rates of self-declared psychiatric issues were descriptively but not statistically compared with available data from previous findings, while for part (b), a series of t-tests was performed to compare symptoms of depression, insomnia, emotion regulation, and social activity between participants with (*n* = 190) and without (*n* = 385) psychiatric issues. Effect sizes were reported as Cohen’s d measures, with the following cut-off values: trivial (ds: 0–0.19), small (ds: 0.20–0.49), medium (ds: 0.50–0.79), or large (ds: 0.80 and greater).

To answer the second, fourth (b), and fifth hypotheses, with a series of Pearson’s correlations we assessed the associations between symptoms of depression, insomnia, emotion regulation, and social activity. 

To answer the third hypothesis, we performed two single t-tests and two X^2^ tests.

To answer the fourth hypothesis (a), we tested with a series of ANOVAs whether and to what extent participants with low, medium, and high cognitive reappraisal differed in their scores for depression, insomnia, emotion suppression, and social activity. For F-tests, effect sizes were indicated in terms of partial eta-squared (η_p_^2^) measures, with 0.01 ≤ η_p_^2^ ≤ 0.059 indicating small, 0.06 ≤ η_p_^2^ ≤ 0.139 indicating medium, and 0.14 > η_p_^2.^ indicating large effect sizes.

To associate symptoms of depression, a multiple regression model was run with the following dimensions: dependent variable: symptoms of depression; predictors: insomnia, emotion regulation, and social activity. The statistical requirements to run a multiple regression model were met [129,130,131]: N = 575 > 100; predictors explained the dependent variables (R = 0.737, R^2^ = 0.543); the number of predictors was 6 (gender, psychiatric issues, insomnia, cognitive reappraisal, emotion suppression, social activity; 10 × 6 = 60 < N (575); and the Durbin–Watson coefficient was 1.82, indicating that the residuals of the predictors were independent. Furthermore, the variance inflation factors (VIFs) were between 1.05 and 1.41: while there are no strict cut-off points for the risk of multicollinearity, VIF < 1 and VIF > 10 indicate multicollinearity [129,130,131].

The level of significance was set at alpha < 0.05. All statistical computations were performed with SPSS^®^, version 29.0 (IBM Corporation, Armonk, NY, USA), for Apple Mac^®^ (Cupertino, CA, USA). 

## 3. Results

### 3.1. General Sociodemographic and Study-Related Information

As shown in Table 1, a total of 575 participants took part in the study. The mean age was 22 years, and the majority of participants were female and bachelor’s students in human medicine. In total, 2 (0.35%) out of 575 indicated that they were of diverse gender.

### 3.2. Prevalence of Self-Declared Psychiatric Issues

A total of 190 participants (33.0%) self-declared that they suffered from psychiatric issues. Table 2 reports the types and frequencies of the psychiatric issues. Descriptively, the prevalence rate was higher compared to the prevalence rates reported elsewhere [30,31]. 

High frequencies were observed for eating disorders, major depressive disorder, anxiety disorders, ADHD, and their combinations.

### 3.3. Symptoms of Depression, Insomnia, Emotion Regulation, and Social Activity among Participants with and without Psychiatric Issues

Table 3 reports the descriptive and inferential statistical indices for depression, insomnia, emotion regulation, and social activity among participants with (n = 190; 33.0%) and without (n = 385; 67%) self-declared psychiatric issues. 

Compared to participants without psychiatric issues, participants with psychiatric issues reported higher scores for depression and insomnia and lower scores for cognitive reappraisal (always with significant *p*-values and small to moderate effect sizes). No descriptively or statistically significant mean differences were observed for emotion suppression. 

### 3.4. Associations between Symptoms of Depression, Insomnia, Emotion Regulation, and Social Activity

Table 4 reports the Pearson’s correlation coefficients, which are not repeated in the text again.

Higher scores for depression were associated with higher scores for insomnia and emotion suppression and with lower scores for cognitive reappraisal and social activity.

Higher scores for insomnia were associated with higher scores for emotion suppression and with lower scores for cognitive reappraisal and social activity.

Higher scores for cognitive reappraisal were associated with higher social activity and were unrelated to emotion suppression.

Higher emotion suppression was associated with lower social activity.

### 3.5. Insomnia Scores (Continuous Dimension) and Categories among Medical Students, Non-Medical Students, and Police Officers

Table 5 reports the descriptive statistics of the Insomnia Severity Index (ISI) scores and categories among medical students, non-medical students, and police officers.

Compared to non-medical students and police officers, the medical students included fewer participants with no insomnia, more with subthreshold insomnia, and more with moderate or severe clinical insomnia (X^2^(N = 1970; df = 6) = 35.75, *p* < 0.001). This pattern was observed even if medical students were compared only to non-medical students (X^2^(N = 1437; df = 3) = 34, *p* < 0.001).

Medical students had statistically significantly higher ISI sum scores (m = 15.51; SD = 5.12) compared to non-medical students (m = 6.56; SD = 4.31; t(1437) = 6.45, *p* < 0.001, d = 0.27) and compared to police officers (m = 6.98; SD = 4.96; t(1106) = 4.28, *p* < 0.001, d = 0.18), though effect sizes were small. 

### 3.6. Categories of Cognitive Reappraisal for Depression, Insomnia, Emotion Suppression, and Social Activity

Dimensions of cognitive reappraisal were categorized into low (n = 106, m = 16.49, SD = 3.44), medium (n = 342, m = 25.89, SD = 2.67), and high (n = 127; m = 34.06, SD = 2.74) cognitive reappraisal and used as independent factors, while depression, insomnia, and social activity were dependent variables. Table 6 reports the descriptive and inferential statistical overview. The higher the category of cognitive reappraisal, the lower the scores for depression and insomnia and the higher the scores for social activity. Scores for emotion suppression did not systematically change as a function of the category of cognitive reappraisal. 

### 3.7. Associations between Symptoms of Depression with Insomnia, Emotion Regulation, Social Activity, Gender, and the Occurrence of Self-Declared Psychiatric Issues in the Regression Model

To associate symptoms of depression, a multiple regression analysis was performed with the following independent variables: insomnia, emotion regulation, social activity, and the categories of psychiatric issues (yes = 1; no = 2) and gender (female = 1; male = 2). 

Table 7 reports the regression model. 

The independent variables explained 54.3% of the variance in depression scores (R^2^ = 0.543; R = 0.74). Higher scores for insomnia, female gender, and occurrence of psychiatric issues and lower scores for social activity predicted higher scores for depression, while dimensions of emotion regulation (cognitive reappraisal; emotion suppression) were excluded from the model, as they did not reach statistical significance. 

## 4. Discussion

The aims of the present study were to assess self-declared psychiatric issues along with symptoms of depression, insomnia, emotion regulation, and social activity among a sample of Swiss medical students. It turned out that 33% reported suffering from self-declared psychiatric issues and that these participants also reported higher scores for depression, insomnia, and emotion suppression and lower scores for cognitive reappraisal and social activity. Further, compared to historical data, the medical students reported more insomnia. Lastly, the occurrence of psychiatric issues, higher insomnia, and lower social activity were strongly associated with higher scores for depression.

The present study expanded upon previous research in the field in the following five ways: First, among Swiss medical students, we assessed concomitantly self-declared psychiatric issues, including symptoms of depression, insomnia, emotion regulation, and social activity, which allowed a more sophisticated and fine-grained analysis of the pattern of associations. Second, the self-declared occurrence of psychiatric issues was related to a broad variety of states of psychological ill-being. Third, compared to non-medical students and professionals working in a high-risk context (police and emergency response service officers), medical students suffered from more insomnia. Fourth, those who reported higher cognitive reappraisal as a proxy of emotion regulation also reported lower scores for depression and insomnia and higher scores for social activity. Fifth, self-declared psychiatric issues, female gender, insomnia, and lower social activity were more strongly and independently associated with higher scores for depression. 

### 4.1. Prevalence Rates of Self-Declared Psychiatric Issues among Medical Students

As described in Section 3.1 and shown in Table 2 and Table 3, prevalence rates of self-declared psychiatric issues were descriptively higher (33%) as compared to meta-analytically reported prevalence rates ([31]: 28%; [30]: 27.2%). To explain this gap, it is important to underline that we asked for the major and most frequent psychiatric issues (always self-declared), such as ADHD, eating disorders, anxiety disorders, substance use disorder, and obsessive–compulsive disorder, while previous meta-analyses focused solely on major depressive disorder [31], including symptoms of depression and suicidal behavior [30]. Thus, we expanded upon previous research in that we asked about self-declared psychiatric issues more broadly and thus reflected the prevalence rates of psychiatric issues among individuals crossing from late adolescence to early adulthood more accurately. 

### 4.2. The Insomnia–Depression Link

Individuals scoring high for insomnia also scored high for symptoms of depression (Table 4), and thus we confirmed what has been extensively observed elsewhere [43,44,45,46,48,49,114]. The novelty here is that this insomnia–depression link was further confirmed among Swiss medical students and that we introduced insomnia as a major confounder for symptoms of depression, while, apparently, previous meta-analytic studies on depression among medical students [30,31] did not consider that higher scores for depression might have been compromised by the occurrence of higher scores for insomnia. 

### 4.3. Insomnia Scores among Swiss Medical Students and Non-Medical Students and Police Officers

Compared to norms for young adult students and police and emergency response service officers, medical students scored higher for insomnia (both continuous and categorical dimensions (see Table 5)). Thus, we confirmed what was observed elsewhere [57,58,59]. However, the novelty is that, in this study, such data were gathered apparently for the first time among Swiss medical students and that these results were put into relation with further important confounders, such as symptoms of depression as well as emotion regulation and social activity (see Table 4). Additionally, given that standardized and tailored psychotherapeutic interventions for insomnia are available and delivered both on-site or online [132,133,134] and both individually or in groups [135,136], Swiss medical schools might consider teaching how to treat and prevent insomnia among their students. 

### 4.4. Emotion Regulation: Cognitive Reappraisal and Emotion Suppression 

As shown in Table 4 and Table 6, those who scored high for cognitive reappraisal also scored higher for psychological well-being, including symptoms of depression, insomnia, and social activity. Thus, we confirmed what has been observed before [94,115,116,117]. The novelty of the results presented here consists in the introduction of the cognitive processes of emotion regulation. Gross described the most comprehensive and evidence-based model of this concept [93,113,115,126,137,138,139,140,141,142,143]. The simplified model consists of the perception of a specific situation and the shifting of attention to the situation, followed by the process of appraisal and response to the situation [94]. It is beyond the scope of the present paper to fully describe Gross’ model of emotion regulation, and we refer to his handbook [94]. For the present study, we focused on cognitive reappraisal and emotion suppression as the two conceptual core strategies of emotion regulation processes related to emotional experience and emotional expression [94,126]. More specifically, the dimension of cognitive reappraisal describes the cognitive–emotional processes of actively and voluntarily modifying the quality of the emotion (e.g., turning a sad feeling into a more cheerful feeling). By contrast, the dimension of suppression relates to the process of actively hindering cognitively the emergence and manifestation of an emotion. Research on these dimensions showed that compared to cognitive reappraisal, emotion suppression was associated with further mental health issues.

In our opinion, similar to the discussion on the treatment of insomnia, cognitive reappraisal might be used as a therapeutic intervention; more specifically, interventions of CBT aim at restructuring dysfunctional cognitive–emotional processes [144,145,146], and it is conceivable that CBT interventions might also decrease symptoms of depression, insomnia, and lack of social activity via cognitive reappraisal techniques among medical students. 

### 4.5. Social Activity and Psychological Well-Being 

As shown in Table 4, those who scored high for social activity also scored low for psychological ill-being, and we confirmed previous results [106,110,111]. The novelty of the present findings consists in the fact that we observed the social activity–psychological well-being link among medical students, that is, among a specific group of future health professionals crossing from late adolescence into early adulthood. 

### 4.6. Independent Dimensions of Sociodemographic and Psychological Dimensions Related to Symptoms of Depression

With the research question, we investigated which sociodemographic and psychological dimensions were more strongly related to symptoms of depression. To this end, we ran a multiple regression analysis (see Table 7). It turned out that female gender, self-declared psychiatric issues, higher scores for insomnia, and lower scores for social activity were more strongly related to symptoms of depression.

As regards the higher prevalence rates of symptoms of depression in females compared to males, [147] noted the following observations: while in childhood, girls are no more depressed than boys, more girls than boys are depressed by ages 13 to 15; and, in adulthood, twice as many women as men are depressed. To understand these prevalence rates, affective (emotional reactivity), biological (genetic vulnerability, pubertal hormones, and pubertal timing and development), and cognitive (cognitive style, objectified body consciousness, and rumination) factors, alongside negative life events, were identified to increase vulnerability to depression among females [147].

In our opinion, the novelty of the present results consists in the following points. First, we observed the described pattern of results among students in general and among medical students in particular, that is, among the future and most responsible healthcare providers for the general population. Second, we were able to identify those students at increased risk for symptoms of depression (females and those reporting self-declared psychiatric issues), including insomnia and decreased social activity. Third, again, since standardized and evidence-based CBT interventions are available for the treatment of symptoms of depression, insomnia, and social anxiety, medical schools might consider offering such interventions as standard care for their students. 

### 4.7. Transdiagnostic Approach

The quality of the data does not allow a deeper understanding of the underlying psychological mechanisms of the present pattern of results. Given this—and what follows is highly speculative—we draw attention to the transdiagnostic approach of psychological ill-being.

In the field of psychiatry and clinical psychology, within the last decade, the *transdiagnostic approach* has gained increased attention as a means of explaining why the effective treatment of a specific psychiatric disorder leads to improvements in further psychiatric issues. To make the case in point, successfully treating symptoms of insomnia also improved symptoms of depression, anxiety, and stress [43,66,136,148,149,150]. To explain this phenomenon, the concept of the *transdiagnostic approach* [66,151,152,153,154,155,156,157,158,159,160] reflects the observation that improvements in one dimension of psychological experience are associated with improvements in further dimensions of psychological experience. Pearl and Norton [154], in their meta-analysis, investigated if and to what extent diagnosis-specific cognitive–behavioral therapy (CBT) was superior to transdiagnostic CBT (tCBT) in the treatment of anxiety disorders. They could not find any clinically significant or statistically meaningful differences between the two treatment approaches. Furthermore, no relationship between comorbidity rates and tCBT outcomes was observed. Likewise, Brand et al. [161] showed that acute bouts of physical activity impacted positively on mood, social interaction, and rumination among inpatients with psychiatric issues, though fully independently of a patient’s psychiatric diagnosis.

### 4.8. Limitations and Future Directions

Despite the novelty of the results, there were some limitations: First, we assessed medical students from a single university, and thus a systematic sample bias cannot be excluded, also because participation was voluntary. Second, we fully relied on self-declared psychiatric issues, while experts’ ratings would have further improved the quality of the pattern of results. Third, by nature, cross-sectional studies preclude the determination of causal relationships between variables. This is a particularly delicate matter when defining a set-up to run multiple regression analyses. Fourth, for methodological reasons, the concept of social activity may have been rather rigid. Indeed, at least three different social interactions have been identified (cf. [162,163,164]: First, while an individual might react to constraints, rules and attitudes in a specific social context (*reactive interaction*), second, the individual also evokes distinctive responses in the social environment (*evocative interaction).* Third, an (adult) individual actively and selectively choses their social environment, or, put the other way round, an individual is not only reactive and evocative in their social environment, but also creates their social world (*proactive interaction*). Such proactive interactions may further shape and impact what an individual believes and how the social world appears to function. With this in mind, it would be interesting to investigate whether and to what extent dimensions of reactive, evocative, and proactive behavior are associated with symptoms of depression and insomnia. Fifth, given that emotional functioning was related to academic achievements [165], it would have been important to understand whether and to what extent dimensions of depression, insomnia, emotion regulation, and social activity patterns were related to the students’ performance during internships and exams. Sixth, by nature, a longitudinal study design would have allowed a more in-depth understanding of the causal relationships between depression, insomnia, emotion regulation, and social activity over time, including the risk of quitting vocational activity as a medical doctor after the achievement of a medical degree [166,167,168]. Lastly, it would have been interesting to experimentally explore whether and under which conditions perceived [107,108] or even subliminally presented social support [109] would have been associated with more favorable scores for psychological well-being. 

## 5. Conclusions

Among a sample of medical students, the prevalence rate of self-declared psychiatric issues was about one-third, and the self-declared psychiatric issues were related to symptoms of insomnia, depression, and dysfunctional emotion regulation, including lower social activity. Given that standardized and evidence-based CBT interventions for depression, insomnia, dysfunctional cognitive–emotional processes, and social anxiety are available, Swiss medical schools might consider offering such interventions to prevent current and future mental health issues.

## Figures and Tables

**Table 1:** Participants’ sociodemographic and study-related characteristics.

Sociodemographic Information	
Total	N = 575
Age, mean (SD)	22.00 (2.44)
Gender	no. (% of n)
Female	396 (68.87)
Male	177 (30.78)
Diverse	2 (0.35)
Study year	no. (% of n)
1. Bachelor	154 (26.78)
2. Bachelor	102 (17.74)
3. Bachelor	86 (14.96)
1. Master	96 (16.70)
2. Master	82 (14.26)
3. Master	55 (9.57)
Study subject	no. (% of n)
Human medicine	543 (94.43)
Dental medicine	32 (5.57)

**Table 2:** Self-declared psychiatric issues based on ICD-10 diagnoses.

Self-Declared Psychiatric Issues				
	Frequency	Percent	Valid Percent	Cumulative Percent
None	385	67.0	67.0	67.0
ADHD	4	0.7	0.7	67.7
Eating disorder	31	5.4	5.4	73.0
MDD	43	7.5	7.5	80.5
Anxiety disorders, including PTSD and OCD	35	6.1	6.1	86.6
ADHD + MDD	7	1.2	1.2	87.8
MDD + eating disorder	16	2.8	2.8	90.6
MOD + anxiety disorder	27	4.7	4.7	95.3
Various psychiatric issues	27	4.7	4.7	100.0
Total	575	100.0	100.0	

Notes: ADHD = attention-deficit/hyperactivity disorder; MDD = major depressive disorder; PTSD = post-traumatic stress disorder; OCD = obsessive–compulsive disorder.

**Table 3:** Descriptive and inferential statistical indices of depression, insomnia, emotion regulation, and social activity among students with (n = 190) and without (n = 385) self-declared psychiatric issues.

	Self-Declared Psychiatric Issues		Statistics	Cohen’s d
	Yes	No		
N	190	385		
	M (SD)	M (SD)		
Depression	10.32 (5.78)	5.78 (4.19)	t(573) = 8.53 ***	0.76 [M]
Insomnia	8.96 (4.81)	7.19 (4.26)	t(573) = 4.47 ***	0.40 [S]
Cognitive reappraisal	24.74 (6.27)	26.56 (6.17)	t(573) = 3.31 ***	0.29 [S]
Emotion suppression	14.72 (4.99)	14.61 (4.92)	t(573) = 0.26	0.02 [T]
Social activity	41.46 (6.72)	45.11 (6.34)	t(573) = 6.34 ***	0.56 [M]

Notes: *** = *p* < 0.001; [T] = trivial effect size; [S] = small effect size; [M] = medium effect size.

**Table 4:** Pearson’s correlation coefficients between depression, insomnia, cognitive reappraisal, emotion suppression, and social activity (N = 575).

Dimensions					
	Depression	Insomnia	Cognitive Reappraisal	Emotion Suppression	Social Activity
Depression	-	0.61 ***	−0.28 ***	0.18 ***	−0.57 ***
Insomnia		-	−0.17 ***	0.14 ***	−0.38 ***
Cognitive reappraisal			-	−0.03	0.33 ***
Emotion suppression				-	−0.27 ***
Social activity					-

Notes *** = *p* < 0.001.

**Table 5:** Descriptive and inferential statistical indices of Insomnia Severity Index (ISI) scores (continuous and categorical dimensions) among medical students, non-medical students, and police officers (historical samples).

Insomnia Severy Index						
		ISI Categories				ISI Sum Score
		No Insomnia	Subthreshold Insomnia	Moderate Clinical Insomnia	Severe Clinical Insomnia	M (SD)
	N	n (%)	n (%)	n (%)	n (%)	
Medical students	575	303 (52.7)	220 (38.3)	46 (8.0)	6 (1.0)	15.51 (5.12)
Non-medical students	864	585 (67.9)	224 (26.0)	49 (5.7)	4 (0.5)	6.56 (4.31)
Police officers	533	331 (62.1)	160 (30.0)	35 (6.6)	7 (1.3)	6.98 (4.96)

**Table 6:** Descriptive and inferential statistical indices of depression, insomnia, emotion suppression, and social activity, recorded separately for categories of cognitive reappraisal.

	Categories of Cognitive Reappraisal			Statistics	
	Low	Medium	High	F-Tests	η_p_^2^
N	106	337	126		
	M (SD)	M (SD)	M (SD)		
Depression	9.58 (5.53)	8.06 (5.13)	6.18 (3.83)	F(2, 572) = 13.82 ***	0.047 [S]
Insomnia	9.21 (5.07)	7.67 (4.32)	6.88 (4.33)	F(2, 572) = 8.08 ***	0.027 [S]
Emotion suppression	14.57 (4.91)	14.87 (4.92)	14.26 (5.02)	F(2, 572) = 0.74	0.003 [S]
Social activity	40.67 (7.41)	43.80 (6.24)	46.87 (5.89)	F(2, 572) = 27.11 ***	0.087 [M]

Notes: *** = *p* < 0.001; [S] = small effect size; [M] = medium effect size.

**Table 7:** Multiple linear regression with depression as the outcome variable and insomnia, social activity, gender, and self-declared psychiatric issues as predictors.

Dimension	Variables	Coefficient	Standard Error	Coefficient β	t	*p*	R	R^2^	Durbin–Watson	VIF
Depression	Intercept	19.87	1.421	-	13.984	<0.001	0.737	0.543	1.818	
	Insomnia Social activity	0.486 −0.257	0.035 0.026	0.436 −0.339	14.036 9.986	<0.001 <0.001				1.184 1.414
	Gender	−1.173	0.321	−0.107	−3.658	<0.001				1.051
	Self-declared psychiatric issue	−1.599	0.326	−0.147	−4.911	<0.001				1.101

Notes: Gender: female = 1; male = 2; self-declared psychiatric issue: yes = 1; no = 2. Excluded variables: cognitive reappraisal and emotion suppression: ts < 1.05; ps > 0.324.

## Data Availability

Data may be made available under the following conditions: 1. An expert in the field asks for data. 2. There must be robust and strong hypotheses to support and justify the request. 3. A statement and proves are needed to make sure that data are securely stored. 4. A statement is needed to make sure that data are not shared with third parties.
